# Chikusetsu Saponin IVa liposomes modified with a retro-enantio peptide penetrating the blood-brain barrier to suppress pyroptosis in acute ischemic stroke rats

**DOI:** 10.1186/s12951-024-02641-y

**Published:** 2024-07-04

**Authors:** Yitong Liang, Tingting Fan, Min Bai, Na Cui, Wangting Li, Jingwen Wang, Yue Guan

**Affiliations:** 1 Department of Pharmacy, Xijing Hospital, Air Force Medical University, Changle West Road 127, Xi’an, Shaanxi China; 2 Department of Geriatrics, Xijing Hospital, Air Force Medical University, Changle West Road 127, Xi’an, Shaanxi China

**Keywords:** Chikusetsu saponin IVa, Liposomes, Acute ischemic stroke, Retro-enantio peptide, THRre-peptide

## Abstract

**Background:**

The therapeutic strategies for acute ischemic stroke were faced with substantial constraints, emphasizing the necessity to safeguard neuronal cells during cerebral ischemia to reduce neurological impairments and enhance recovery outcomes. Despite its potential as a neuroprotective agent in stroke treatment, Chikusetsu saponin IVa encounters numerous challenges in clinical application.

**Result:**

Brain-targeted liposomes modified with THRre peptides showed substantial uptake by bEnd. 3 and PC-12 cells and demonstrated the ability to cross an in vitro blood-brain barrier model, subsequently accumulating in PC-12 cells. In vivo, they could significantly accumulate in rat brain. Treatment with C-IVa-LPs-THRre notably reduced the expression of proteins in the P2RX7/NLRP3/Caspase-1 pathway and inflammatory factors. This was evidenced by decreased cerebral infarct size and improved neurological function in MCAO rats.

**Conclusion:**

The findings indicate that C-IVa-LPs-THRre could serve as a promising strategy for targeting cerebral ischemia. This approach enhances drug concentration in the brain, mitigates pyroptosis, and improves the neuroinflammatory response associated with stroke.

**Supplementary Information:**

The online version contains supplementary material available at 10.1186/s12951-024-02641-y.

## Graphical Abstract

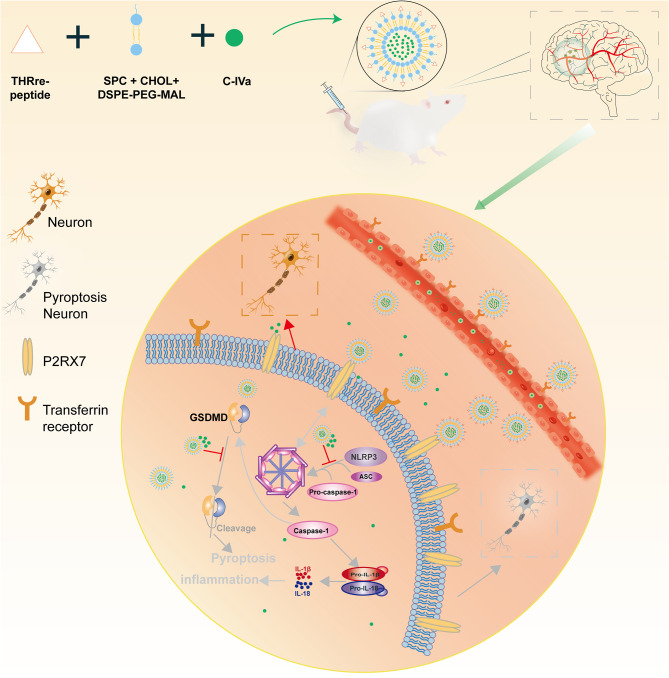

## Introduction

Acute ischemic stroke (AIS) leads to neuronal injury and has become the second most prevalent cause of disability globally, with a rising mortality rate each year [1–3]. Conventional treatments focus on improving cerebral blood circulation but are limited by numerous therapeutic disadvantages [4]. Recently, neuroprotectants have garnered significant attention as an alternative stroke therapy, offering the potential to directly target brain parenchyma during AIS and improve patient outcomes.

Research has indicated that brain cells in the ischemic penumbra remain viable post-stroke [5], but without rapid restoration of circulation, secondary neuroinflammation can lead to cell death [6]. Pyroptosis, a recently recognized form of cell death, is increasingly linked to various inflammation-related conditions and is a significant pathogenic factor in stroke, making it a prominent research focus and target for therapeutic intervention [7, 8]. Studies have highlighted neuroinflammation as a critical factor in stroke prognosis, showing that curbing the inflammatory response post-AIS prevents brain damage and enhances long-term neurological outcomes. Additionally, reperfusion injury, whether through self-perfusion without thrombolysis or post-thrombolytic reperfusion, poses a risk of irreversible neuronal damage [9]. Therefore, safeguarding neurons during ischemia-reperfusion to aid functional recovery is crucial. However, the blood-brain barrier’s (BBB) high selectivity impedes many potential CNS treatments, presenting a significant challenge in the therapy for stroke [10, 11]. 

Chikusetsu saponin IVa (C-IVa), primarily obtained from the dried rhizome of *Panax japonicus* C.A. Mey., is also present in other plants like Araliaceae and Congmunmunia Araliaceae. Recently, it has shown significant activity in combating diabetes [12], lipotoxicity [13], and cardiomyopathy [14]. Regarding neuroprotection, C-IVa has demonstrated its ability to inhibit apoptosis, inflammation, and oxidative stress in diabetic stroke mice through AMPK-mediated GSK-3β phosphorylation within the APN-LKB1 pathway [15]. C-IVa has been found to offer neuroprotection by inhibiting the NLRP3/Caspase-1 pathway and enhancing SIRT1/ERK1/2 levels [16, 17]. This suggests that C-IVa could be a promising neuroprotective agent, potentially effective against AIS by preventing pyroptosis. However, in practical application, due to its high molecular weight and water solubility, molecular weight and water solubility, C-IVa faces challenges in crossing the BBB to reach ischemic regions, thus, it was necessary to modify its dosage form to enhance its therapeutic efficacy.

Liposomes was selected as our drug delivery system due to their biocompatibility and effectiveness in enhancing drug accumulation in the brain [18, 19]. And a retro-enantio peptide, THRre-peptide (Cpwvpswmpprht-NH2), was selected as the targeting “warhead”, which was derived by Prades R et al. [20] using a retro-enantio approach based on the THR peptide (THRPPMWSPVWP). Because THRre peptide has almost the same topochemical shape as THR peptide, it can also target the transferrin receptor (TfR). Studies by Prades R et al. showed that THRre-peptide, when attached to nano-preparations, exhibited greater stability in serum and higher protease resistance compared to THR peptide due to the inability of proteases to recognize D-type amino acids, enhancing BBB penetration [20, 21]. Arranz-Gibert P et al. further confirmed that THRre-peptide was less immunogenic than its parent peptide [22]. Subsequently, Bukchin A et al. attached THRre peptide to amphiphilic polymeric nanoparticles, and verified that it can improve the performance of nanoparticle shuttle BBB [23]. Meanwhile, TfR, as a specific target for THRre peptide, was abundantly expressed in cerebral microvasculature [24] and is more prevalent on the BBB than other receptors such as LDL receptor [25]. In addition, THRre peptide is non-toxic and does not compete with transferrin [20], making it a promising strategy for BBB penetration due to these combined advantages.

Therefore, we aimed to prepare THRre-peptide-carrying C-IVa liposomes to utilize the specificity of the target peptide binding to the receptor to efficiently deliver the drug to ischemic injury sites, and to exert the cerebral neuroprotective effects of C-IVa in anti-inflammation and inhibition of pyroptosis.

## Materials and methods

### Materials

THRre-peptide (sequence: Cpwvpswmpprht-NH2) was obtained from Qiang Yao Biotechnology Co., Ltd. (Shanghai, China). 1,1’-Dioctadecyl-3,3,3’,3’-Tetramethylindotricarbocyanine Iodide (DIR), DSPE-PEG_2000_, and DSPE-PEG_2000_-Mal were supplied by Xi’an Ruixi Biological Technology Co. Ltd. (Xi’an, China). Phosphatidylcholine and Cholesterol were purchased from Rhawn Chemical Technology Co. Ltd. (Shanghai, China). 3-(2-Benzothiazolyl)-N, N-diethylumbelliferylamine (Coumarin 6) was purchased from Macklin Biochemical Technology Co., Ltd. (Shanghai, China).

### Fabrication and characterization of liposomes

#### Synthesis and characterization of DSPE-PEG_2000_-THRre

THRre-peptide and DSPE-PEG_2000_-Mal were conjugated through a sulfhydryl-maleimide coupling method. DSPE-PEG_2000_-Mal was dissolved in acetonitrile while THRre-peptide was dissolved in PBS (pH = 7.4). The solutions were stirred together at room temperature for 12 h under nitrogen protection (1.5:1, molar ratio). After the reaction was completed, deionized water was dialyzed (MWCO 3.5 kDa) for 48 h to remove excess acetonitrile. The product was characterized by NMR hydrogen spectroscopy (^1^H-NMR) and MALDI-TOF MS.

#### Preparation and characterization of liposomes

Liposomes were prepared by the thin-film hydration method. The unmodified liposomes were composed of SPC/Cholesterol/DSPE-PEG_2000_ (15/5/5, w/w/w) and the THRre-peptide-modified liposomes consisted of SPC/Cholesterol/DSPE-PEG_2000_-THRre (15/5/5, w/w/w). Both types of liposome components were dissolved in methylene chloride and formed into films by vacuum evaporation at 25 °C. The resulting dry films were hydrated with PBS containing 4 mg of C-IVa at 55 °C for 2 h, followed by ultrasonic treatment to reduce particle size and centrifugation to eliminate free C-IVa. Fluorescent dye-labeled liposomes were similarly prepared using Coumarin 6 (COUR 6) or DiR dye.

Zetasizer Nano ZSE system (Malvern Instruments Ltd., Malvern, UK) determined the ζ-potential, particle size, and polydispersity index (PDI) of the liposomes. The concentration of free C-IVa was examined by high-performance liquid chromatography (HPLC; Shimadzu, Japan) at a detection wavelength of 203 nm [26–28]. The encapsulation efficiency (EE) was calculated with the formula: EE % = (total C-IVa - free C-IVa) /total C-IVa × 100%. Drug loading (DL) capacity was determined using the equation: DL % = (total C-IVa - free C-IVa) /liposome preparation × 100%. The HPLC eluent for C-IVa was a mixture of 65% acetonitrile and 35% H_2_O with 0.1% phosphoric acid.

The morphology of liposomes was observed by transmission electron microscope (TEM, HITACHI Ltd., Japan). 0.1 mg/ml solution of either unmodified or THRre-peptide-modified liposomes was applied to a 200 mesh copper grid and allowed to sit for 2 to 3 min, and the excess liquid was absorbed with filter paper. 2% phosphotungstic acid staining for 1 min and then absorbed the excess staining solution. The liposome morphology was then observed under the TEM.

Dialysis assay detected liposome release behavior in vitro. 1mL liposome solution was placed in a dialysis bag (MWCO 1.0 kDa) and submerged in a centrifuge tube containing 35 mL of PBS (pH 7.4). Oscillating at 80 RPM at 37 °C, samples of 1 mL were collected at 0.5, 1, 2, 4, 6, 8, 12, 24, and 48 h, and the C-IVa content was analyzed using HPLC. After each sampling, 1 mL of fresh PBS was added to maintain the volume.

Liposome stability was assessed by analyzing the particle size and encapsulation efficiency of liposome solutions on the 1st, 3rd, 5th, and 7th days of a week.

#### Evaluation of liposome biosafety in vitro

The biosafety was evaluated by hemolysis test and cytotoxicity test in vitro.

For the hemolysis test, 10 mL of fresh rat blood was stirred with a glass rod to remove fibrin, then diluted with saline at a 1:5 ratio. After centrifugation at 1500 rpm for 15 min, the supernatant was discarded, and the erythrocytes were washed 3 to 4 times with saline until the supernatant was clear. The resulting erythrocytes were suspended to a 2% concentration. The experiment was divided into a saline group (negative control), C-IVa group, C-IVa-LPs group, C-IVa-LPs-THRre group, and 1% Triton X-100 group (positive control). Each group received 2.2 mL of the 2% erythrocyte suspension and 0.3 mL of the test solution, followed by incubation at 37 °C for 3 h. Post-incubation, the samples were centrifuged, and the OD value of the supernatant was measured to calculate the hemolysis rate [21].

Cytotoxicity of the liposomes was evaluated using a CCK-8 kit. bEnd. 3 or PC-12 cells were seeded in 96-well plates at a density of 1 × 10^4^ cells per well and cultured in DMEM high-glucose medium containing 10% fetal bovine serum and 1% penicillin/streptomycin at 37 °C with 5% CO_2_. After 24 h, cells were treated with C-IVa, C-IVa-LPs, or C-IVa-LPs-THRre at concentrations of 10, 20, 40, 60, 80, 100, and 120µM. Following another 24 h of incubation, 100 µL of CCK-8 solution (10 µL stock solution and 90 µL DMEM) was added to each well and incubated for 2 h. Absorbance was then measured at 450 nm using a MicroplateReader (Thermo, USA).

*In vivo, safety was assessed by measuring plasma levels of alanine aminotransferase (ALT), aspartate aminotransferase (AST), urea (BUN), and creatinine (CREA), and observing the injury of the heart, liver, spleen, lung, and kidney through hematoxylin and eosin (H&E) staining. Blood samples were collected from the Control group, MCAO group, MCAO + C-IVa group, MCAO + C-IVa-LPs group, and MCAO + C-IVa-LPs-THRre group groups into heparinized tubes, and plasma was obtained by centrifugation and analyzed using appropriate kits for ALT, AST, BUN, and CREA (Nan jing jiancheng, China).*

### Establishment of OGD/R

The OGD/R models of bEnd.3 cells [29] and PC-12 cells [30] were constructed according to the previous methods. After normal culture conditions, the high-glucose medium containing serum was replaced by the glucose-free, serum-free medium. Then the cells were placed in a hypoxia incubator chamber (37°C, 95% N_*2 *_and 5% CO_2_), bEnd.3 cells were cultured for 2 h and PC-12 cells for 4 h. After this period, the medium was replaced with a high-glucose medium containing the test drug, and the cells were reoxygenated in a CO₂ incubator for 24 h (37°C, 95% air, and 5% CO_2_).

### PC-12 cell viability assessment

At the same time of cell reperfusion, PC-12 cells were treated with C-IVa, C-IVa-LPs, and C-IVa-LPs-THRre, which were converted into C-IVa-containing 40 µM. Cell viability was assessed 24 h post-treatment using a CCK-8 kit.

### Cellular uptake

bEnd.3 cells and PC-12 cells were inoculated in glass dishes at a density of 3 × 10^5^ and cultured at 37 °C and 5% CO_2_ for 24 h. A 10 μM concentration of Coumarin-6 liposome solution was prepared in the medium, and cells were incubated for 0.5 h and 2 h. After incubation, cells were washed three times with PBS for 10 min each. Following fixation with paraformaldehyde, cells were washed with PBS three times for 5 min each. DAPI staining was then performed for 15 min, followed by three washes with PBS. Cells were observed using a confocal laser scanning microscope (CLSM). In parallel, cells were seeded in six-well plates at the same density and treated identically. Finally, cells were collected for quantitative analysis a flow cytometer (FCM) (Beckman, USA).

### Targeting delivery studies using in vitro BBB model

The construction of BBB model was based on established protocols [31, 32]. bEnd.3 cells (1 × 10^5^) were inoculated in the donor chamber of 24-well transwell (8 μm mean pore size, Corning, USA), and cultured in DMEM high-glucose medium containing 10% fetal bovine serum and 1% penicillin/streptomycin at 37 °C with 5% CO_*2*_. Transendothelial electrical resistance (TEER) was used to determine the integrity of monolayer cells. When the TEER value exceeded 200 Ω/cm^*2*^, liposomes containing coumarin 6 (10 µg/mL) were added. After a 6-h incubation, the solution from the lower chamber was analyzed using fluorescence.

PC-12 cells were inoculated in recipient chamber of the 24-well transwell. After incubation for 1 h and 6 h, the lower PC-12 cells were collected for qualitative analysis using CLSM and quantitative detection via FCM.

### Establishment of animal model

The experimental protocol was approved by the Experimental Animal Ethics Committee and and conducted according to the the Air Force Medical University Animal Experimental guidelines. To establish the middle cerebral artery occlusion (MCAO) model in rats [33, 34], anesthetized rats were placed in a supine position. A midline neck incision of approximately 3–4 cm was made to separate the muscle layer and expose the right common carotid artery (CCA), internal carotid artery (ICA), and external carotid artery (ECA). The ECA and proximal part of the CCA were ligated, and a slipknot was placed on the ICA. A triangular opening was made above the CCA, and a treated nylon thread was inserted into the ICA and middle cerebral artery. After 2 h of occlusion, the thread was removed to allow reperfusion, the wound was sutured, and drugs were administered via tail vein injection. Neurofunctional assessments were performed 24 h post-administration, followed by anesthesia and brain sectioning. 2,3,5-triphenyl − 2 H-Tetrazolium Chloride (TTC) staining was used to evaluate the degree of infarction. A total of 100 adult male SD rats, weighing approximately 250 g to 300 g, were randomly divided into the Control group, MCAO group, MCAO + C-IVa group, MCAO + C-IVa-LPs group, and MCAO + C-IVa-LPs-THRre group.

### Assessment of neurological deficits

The neurological function of MCAO rats was assessed using the Z-Longa scor [35], which ranges from 0 to 5: 0 indicates no neurological deficits; 1 signifies adduction flexion of the contralateral forelimb during tail lifting; 2 denotes rotation to the opposite side during crawling; 3 is for leaning sideways when standing or crawling; 4 represents no voluntary movement and disturbance of consciousness; and 5 indicates death.

### H&E staining

The brain tissue was isolated and fixed in 4% paraformaldehyde. After fixation, conventional paraffin embedding and section were performed, and the sections were soaked in 40 °C warm water to fully stretch the tissue. Then dewaxed with xylene, soaked in ethanol to wash away xylene, and finally stained with hematoxylin and eosin. After dyeing, the samples were air-dried and sealed, observed under an inverted microscope, and photographed.

### In vivo living fluorescent imaging

In vivo, fluorescence imaging of SD rats was conducted to evaluate liposome penetration of the BBB. The rats were divided into three groups: DiR, DiR-LPs, DiR-LPs-THRre. Fluorescence distribution in the brain was observed using the IVIS imaging system at 1 h, 2 h, 4 h, 8 h, 12 h, and 24 h after tail vein injection. After the 24-hour observation period, the rats were sacrificed, and the brain, heart, liver, spleen, lungs, and kidneys were collected for ex vivo analysis. After the completion of the study, the brain tissue was fixed in paraformaldehyde and prepared for frozen sectioning to examine drug distribution.

### Immunofluorescence (IF)

24 h after administration, rats were anesthetized with sodium pentobarbital (30 mg/kg). Brain tissues were isolated, fixed in 4% paraformaldehyde for 24 h, and then embedded in paraffin for sectioning. The sections were incubated overnight at 4°C with anti-GSDMD (Wanlei, WL05686, 1:200), anti-Caspase-1 (Proteintech, 22915-1-AP, 1:100), anti-NLRP3 (Affinity, DF7438, 1:200) and anti-ASC (Abclonal, A11433, 1:200) dilutions. After rewarming, they were incubated with a secondary antibody for 1 h at room temperature in the dark. Finally, DAPI working solution was added dropwise, and the sections were sealed for microscopic observation and image acquisition.

### Cytokine measurement

ELISA kits were employed to measure the concentrations of cytokines IL-1β and IL-18 (Elabscience, China).

### Western blot analysis

Drug-treated PC-12 cells and brain tissues from ischemic penumbra cortices were collected for homogenization using radioimmunoprecipitation assay RIPA lysate containing protease and phosphatase inhibitors. The samples were lysed on ice for 30 min, and centrifuged at 12,000 rpm for 20 min at 4 °C, and the protein concentration in the supernatant was determined using a Protein Assay Kit (NCM Biotech, China). Equal amounts of the protein samples were separated by electrophoresis with 12% SDS-PAGE gels and then transferred onto a polyvinylidene difluoride membrane (PVDF). The membranes were incubated with antibodies targeting P2RX7 (Abclonal, A10511, 1:500), GSDMD (Wanlei, WL05686, 1:500), Caspase-1 (Proteintech, 22915-1-AP, 1:500), NLRP3 (Affinity, DF7438, 1:500), ASC (Abclonal, A11433, 1:500) and β-actin (Servicebio, GB15003, 1:2000) at 4 °C overnight. After washing with PBST (3 times, each for 10 min), the membranes were incubated with secondary antibodies for 1 h at room temperature. Protein detection was performed using the ECL method and visualized with a gel imaging system (Bio-Rad, USA).

### Statistical analysis

Data were analyzed using Prism software and presented as Mean ± SD. Statistical comparisons between different groups were analyzed by One-way analysis of variance (ANOVA). Liver and renal function measurements and neurological deficit scores were expressed as medians. For all the data, *P* < 0.05 was considered to be statistically significant.

### Result

#### Synthesis and characterization of DSPE-PEG_2000_-THRre

**Fig. 1:**
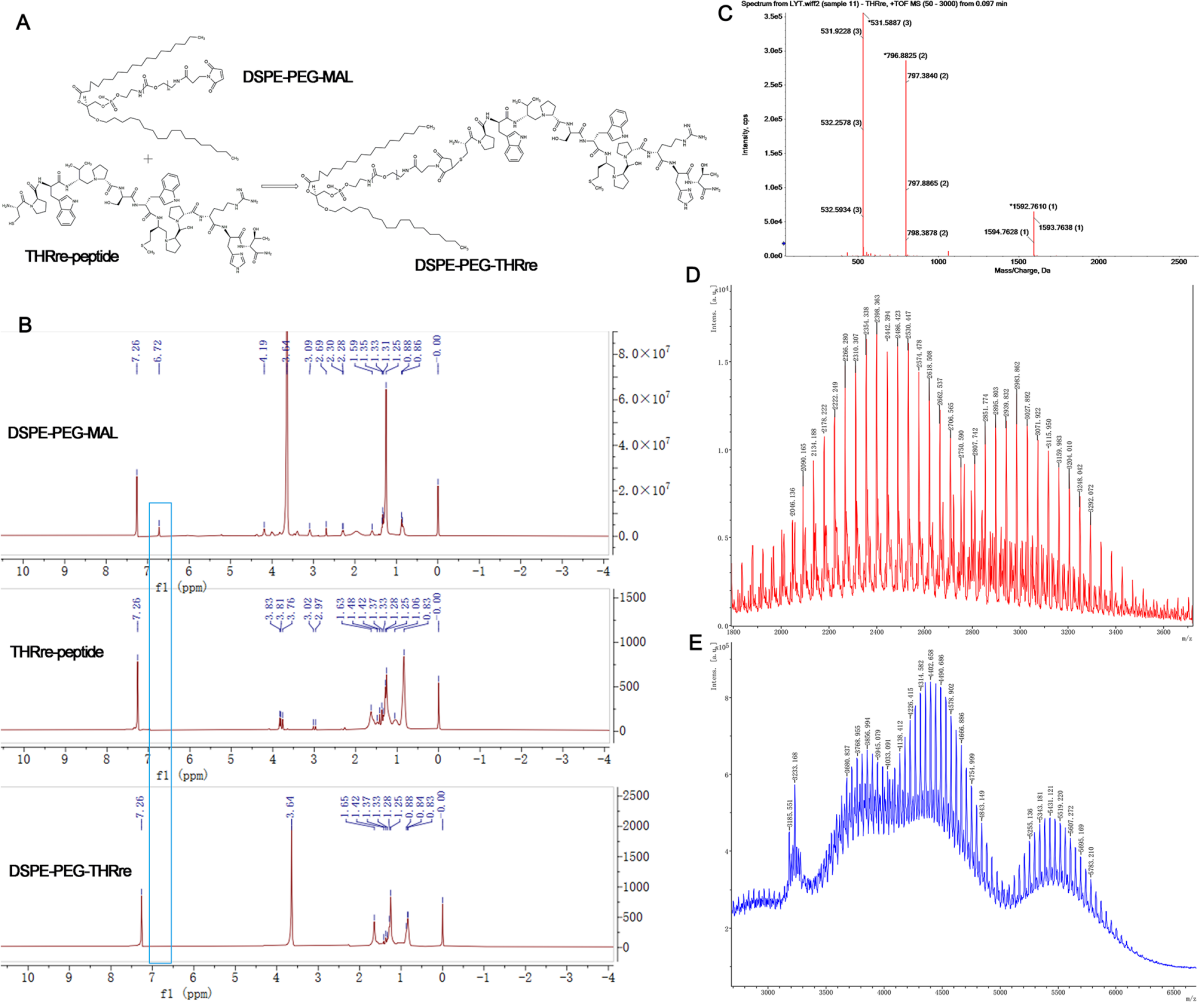
Characterization of DSPE-PEG-THRre. (**A**) Scheme of chemical synthesis preparation of DSPE-PEG-THRre. (**B**) The ^1^H-NMR results of DSPE-PEG-THRre. (**C**) The MALDI-TOF mass spectrum of THRre-peptide. (**D**) The MALDI-TOF mass spectrum of DSPE-PEG-Mal. (**E**) The MALDI-TOF mass spectrum of DSPE-PEG-THRre

The synthesized DSPE-PEG-THRre was verified by ^1^H-NMR and MALDI-TOF-MS (as shown in Fig. 1). The ¹H-NMR spectrum indicated the presence of a signal at 6.72 ppm for the Mal group in DSPE-PEG-Mal, which disappeared in the DSPE-PEG-THRre spectrum. Meanwhile, the chemical shifts of the DSPE and PEG characteristic peaks were 3.64 ppm and 1.25 ppm, respectively, and they were still present in the DSPE-PEG-THRre mapping, indicating successful synthesis. In the MS analysis, the molecular weight (m/z) of DSPE-PEG-Mal ranged from 2000 to 3400, and the m/z of THRre-peptide was 1592.87. A rightward shift in the peak after synthesis further indicated the successful formation of DSPE-PEG-THRre.

#### Preparation and characterization of liposomes

**Fig. 2:**
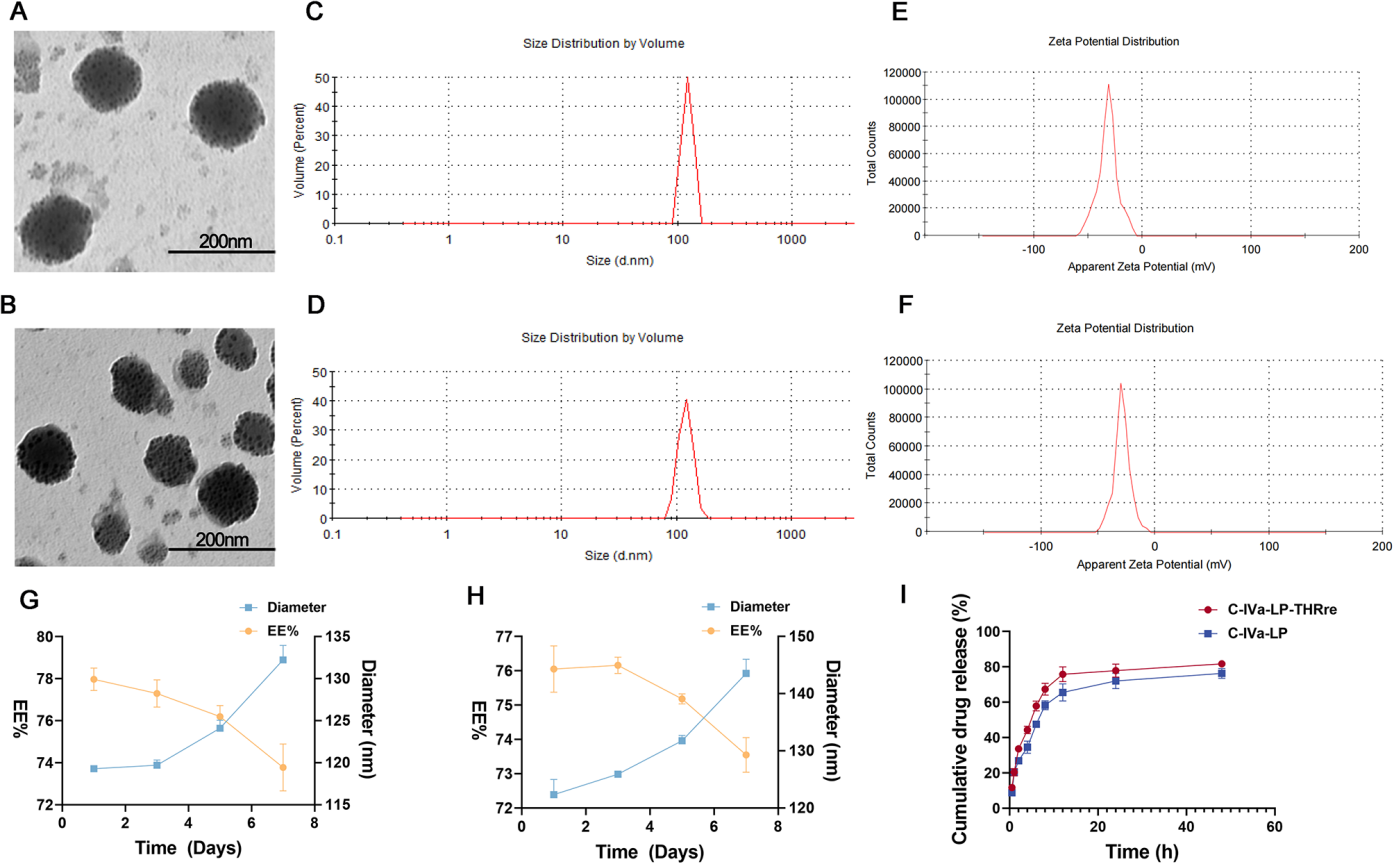
Liposome characterization. (**A**) TEM images of C-IVa-LPs (scale bar = 200 nm). (**B**) TEM images of C-IVa-LPs-THRre (scale bar = 200 nm). (**C**) Particle size distribution of C-IVa-LPs. (**D**) Particle size distribution of C-IVa-LPs-THRre. (**E)** Zeta potential distribution of C-IVa-LPs. (**F)** Zeta potential distribution of C-IVa-LPs-THRre. (**G)** Stability of C-IVa-LPs (*n* = 3). (**H)** Stability of C-IVa-LPs-THRre (*n* = 3). (**I)** The cumulative C-IVa release from C-IVa-LPs and C-IVa-LPs-THRre in PBS solution (*n* = 3)

Two different types of liposomes were prepared using the thin-film hydration method. The TEM plots (Fig. 2A&B) image visualized the appearance and morphology of the liposomes, both of which are spherical and both smaller than 200 nm. Compared with blank liposomes (Supplementary Fig. 1), the C-IVa-coated liposomes exhibited less morphological regularity. As shown in Fig. 2C&D, dynamic light scattering data analysis showed that C-IVa-LPs and C-IVa-LPs-THRre had the particle size of 119.3 ± 0.21 nm and 122.3 ± 2.65 nm, respectively, with zeta potentials of -25.4 ± 0.95 mV and − 20.5 ± 3.66 mV *(*Fig. 2D&E). The particle size of blank liposomes was 93.33 ± 0.46 nm, which increased after loading C-IVa. EE and DL values indicated good efficiency of liposomes loading of C-IVa, both shown in Supplementary Table 1. Liposome stability, assessed by changes in particle size and EE% over 7 days, is presented in Fig. 2G&H. Figure 2I *demonstrates the in vitro release rates of liposomes in PBS. The drug release behavior of the two types of liposomes was similar, and the 48-hour release rate for C-IVa-LPs was below 80%, while for C-IVa-LPs-THRre, it was around 80%.*

#### Evaluation of liposome biosafety

**Fig. 3:**
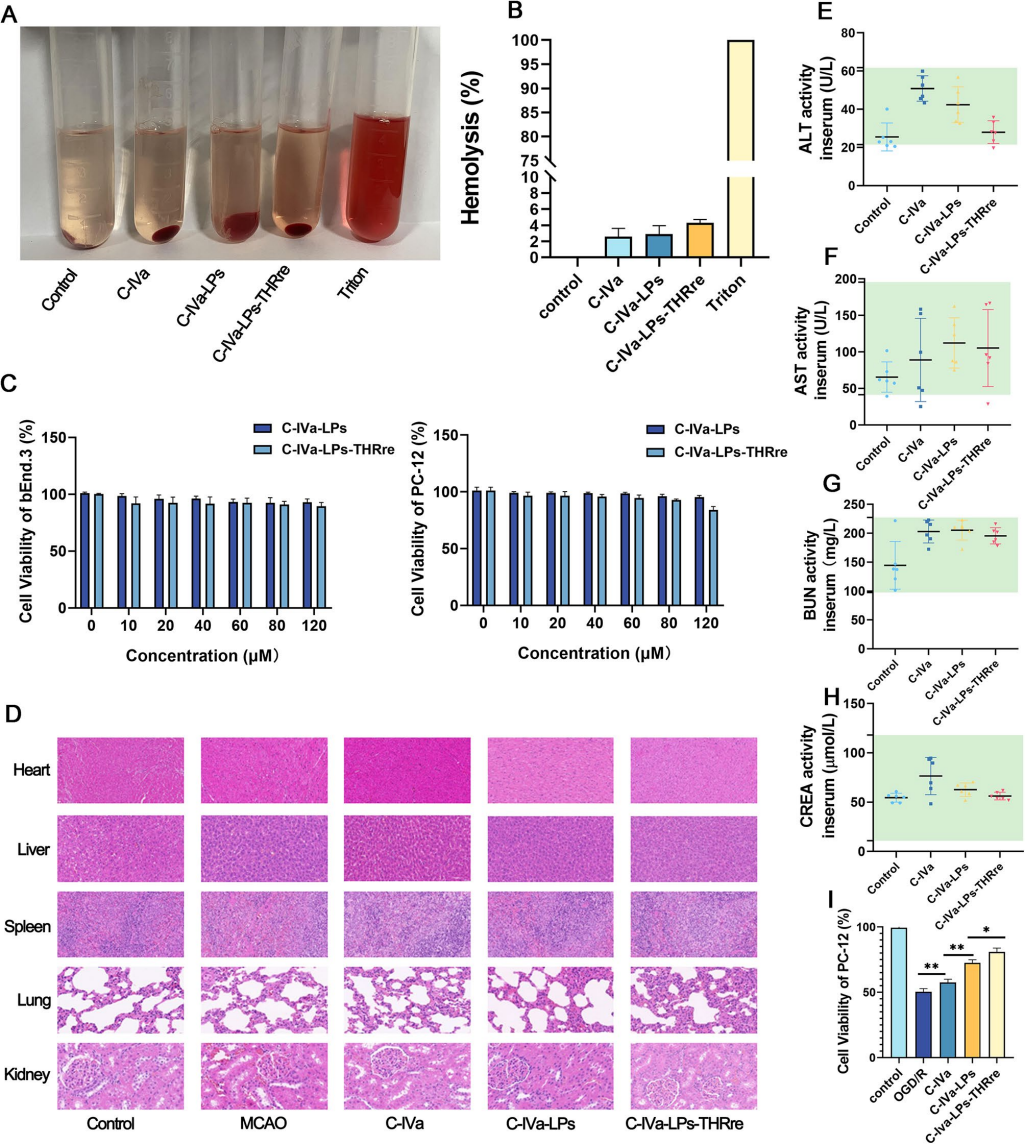
Safety evaluation of C-IVa-LPs and C-IVa-LPs-THRre. (**A**) Disruption of erythrocyte membranes by C-IVa, C-IVa-LPs and C-IVa-LPs-THRre. (**B**) Hemolytic activity of C-IVa, C-IVa-LPs and C-IVa-LPs-THRre (*n* = 3). (**C**) Toxic effects of C-IVa-LPs and C-IVa-LPs-THRre at different concentrations on bEnd.3 and PC-12 cells (*n* = 6). (**D**) H&E staining of brain, heart, liver, spleen, lung, and kidney tissue of MCAO rats (scale bar = 100 μm). **E-H**: Effect of C-IVa, C-IVa-LPs and C-IVa-LPs-THRre on ALT, AST, BUN and CREA activity in serum of MCAO rats. Green background indicates normal range (*n* = 6). (**I)** Cell viability of PC-12 cells (*n* = 6, ** *P* < 0.01; * *P* < 0.05)

The hemolysis assay was conducted to evaluate the safety of liposome intravenous injection in vitro [36]. As shown in Fig. 3A&B, at the injection dose of 10 mg/kg, the hemolysis rate of all groups was below 5%. This result indicates that the two types of liposomes are hemocompatible and suitable for intravenous use. The cytotoxicity results, depicted in Fig. 3C, reveal that both liposomes exhibited slight toxicity to PC-12 and bEnd. 3 cells. C-IVa-LPs-THRre was slightly more toxic than C-IVa-LPs, but cell survival rates remained above 80% across the tested concentrations.

*The in vivo safety of liposomes was assessed by examining the heart, liver, spleen, lungs, and kidneys for damage using H&E staining. Plasma from MCAO rats treated with the drugs was also collected to measure AST, ALT, BUN, and CREA levels.* Fig. 3D displays the H&E stained sections of organ tissues from MCAO rats. No significant differences were observed between the liposome-treated groups, the C-IVa-treated group, and the control group. As shown in Fig. 3E-H, the plasma AST, ALT, BUN, and CREA values of rats in all groups were within normal values.

### PC-12 cell viability assessment

To validate the effect of liposomes on cell viability in PC-12 cells post-OGD/R treatment, a concentration of 40 µM (containing C-IVa) was selected for cellular delivery. As shown in Fig. 3I, *compared with the C-IVa administration group, the cell survival rate was significantly increased after treatment with two types of liposomes respectively. Notably, the cell survival rate in the C-IVa-LPs-THRre group was significantly higher than in the C-IVa-LPs group. Therefore, the results indicate that the C-IVa-LPs-THRre group can markedly reverse OGD/R-induced damage and improve cell survival.*

### Cellular uptake

**Fig. 4:**
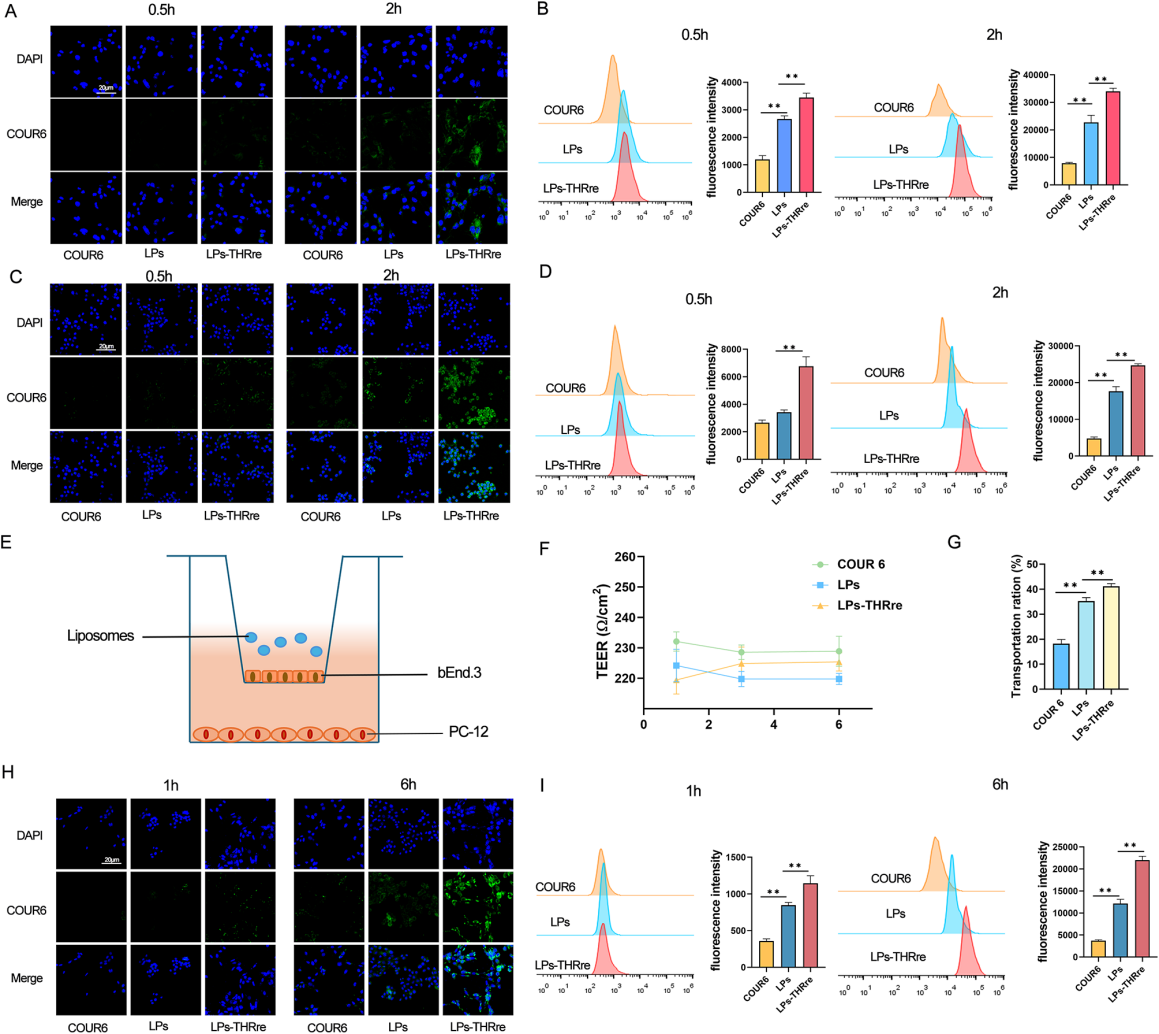
Efficiency of liposome in cell uptaking and blood-brain barrier penetration. (**A**) The uptake of COUR6 labeled liposomes in OGD/R treated bEnd.3 cells was assayed by CLSM (scale bar = 20 μm). (**B**) The typical FCM diagram and statistical analysis of fluorescence intensity of COUR6 in bEnd.3 cells for 0.5 to 2 h (n = 3, ** *P*＜0.01, * *P*＜0.05). (**C**) The uptake of COUR6 labeled liposomes in OGD/R treated PC-12 cells was assayed by CLSM (scale bar = 20 μm). (**D**) The typical FCM diagram and statistical analysis of fluorescence intensity of COUR6 in PC-12 cells for 0.5 to 2 h (n = 3, ** *P*＜0.01, * *P*＜0.05). (**E**) Schematic diagram of BBB model in vitro. (**F**) TEER value changes within 6 h (n = 3). (**G**) The penetration efficiency of LPs-THRre across the BBB (n = 3, ** *P*＜0.01, * *P*＜0.05). (**H**) Uptake of COUR6-loaded liposomes by PC-12 cells was analyzed using LCM in vitro BBB model (scale bar = 20 μm). (**I**) The typical FCM diagram and statistical analysis of fluorescence intensity of COUR6 in vitro BBB model (*n* = 3, ** *P* < 0.01; * *P* < 0.05)

Coumarin-6 was utilized as a fluorescent probe to compare the uptake efficiency of two liposomes and free Coumarin-6 on *OGD/R treated* bEnd.3 and PC-12 cells. As shown in Fig. 4A&C, CLSM observation visualized that the liposomes containing THRre-peptide showed significantly higher fluorescence intensity than free coumarin and unmodified liposomes in both types of cells. Additionally, the fluorescence accumulation in the cells increased significantly over time. Figure 4B&D present the flow cytometry quantification results of liposome uptake in both types of cells. Consistent with the confocal microscopy observations, the THRre peptide modification notably enhanced liposome uptake, with the efficiency being time-dependent.

### Targeting delivery studies using in vitro BBB model

The effects of different liposomes penetrating the BBB in vitro were compared by using transwell assays (Fig. 4E). As shown in Fig. 4F, COUR 6, LPs, and LPs-THRre had no significant effect on TEER values within 6 h, indicating that the BBB integrity remained intact. Figure 4G *illustrates the efficiency of liposome penetration through the BBB within this time frame, with LPs-THRre demonstrating superior penetration. Then we studied the uptake efficiency of liposomes in PC-12 cells in the lower chamber.* As shown in Fig. 4H, CLSM results indicated that the fluorescence signal of THRre peptide-modified liposomes was significantly stronger in PC-12 cells than in other groups. Flow cytometry measurements of intracellular fluorescence intensity confirmed these findings (Fig. 4I). These results suggest that THRre-peptide enhances BBB penetration and promotes liposome accumulation in the brain.

### Mechanism of C-IVa-LPs-THRre anti-pyroptosis in vitro

**Fig. 5:**
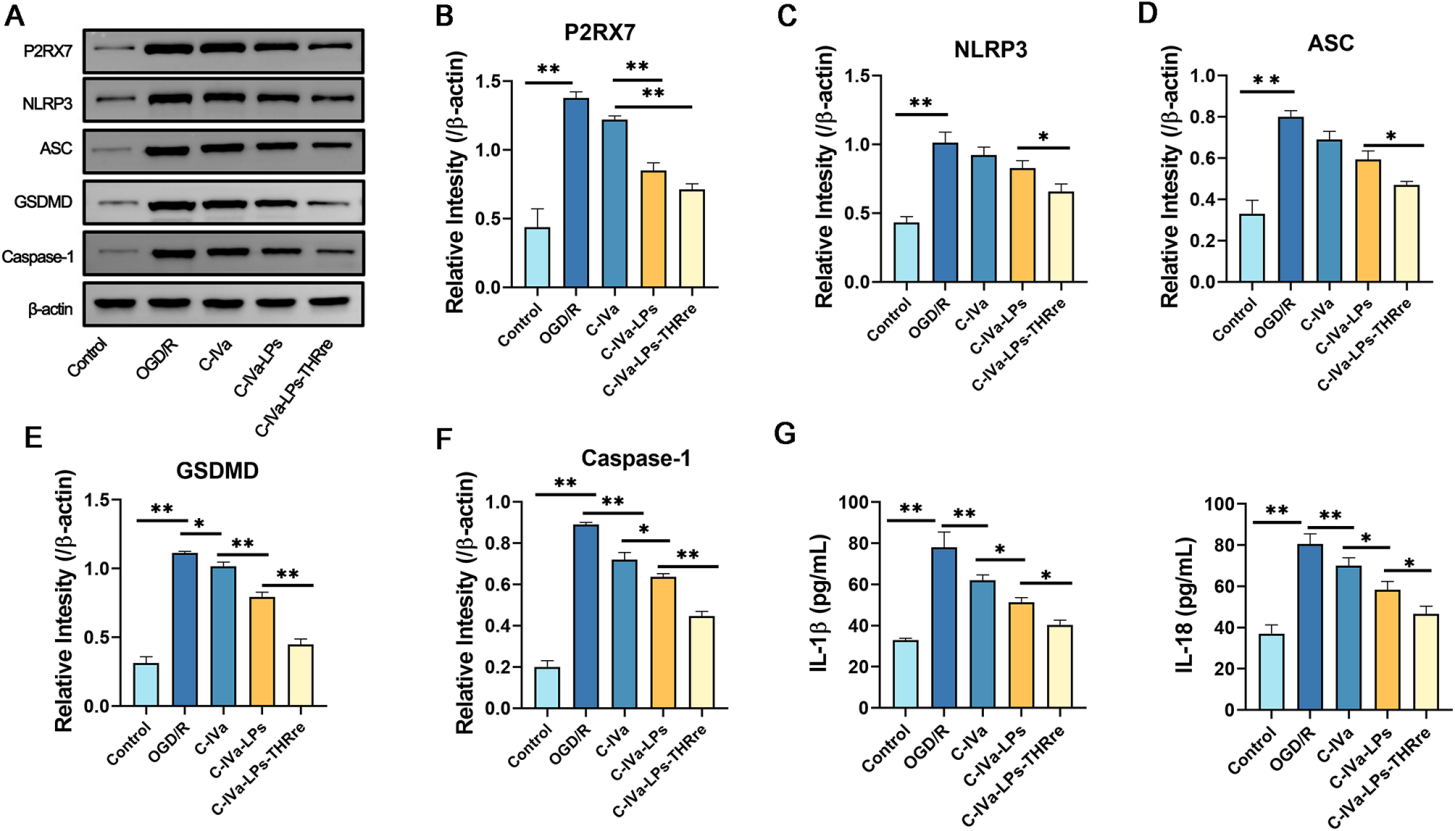
Effect of C-IVa-THRre on expression of pyroptosis related protein in PC-12 cells treated by OGD/R. **A.** The expression levels of P2RX7, NLRP3, ASC, GSDMD and Caspase-1 were detected by Western blot. **B-F**. Statistical analysis in the expression levels of P2RX7, NLRP3, ASC, GSDMD and Caspase-1. **G.** Effects of C-IVa-LPs-THRre on the expression levels of IL-1β and IL-18. (*n* = 3, ** *P* < 0.01; * *P* < 0.05)

The influence of C-IVa-LPs-THRre liposomes on NLRP3 inflammasome-mediated pyroptosis was explored by developing an OGD/R model in PC-12 cells. As shown in Fig. 5, both liposomes markedly decreased the expression levels of P2RX7, NLRP3, ASC, caspase-1, and GSDMD proteins, compared to control PC-12 cells treated with free C-IVa. Notably, liposomes modified with the THRre peptide demonstrated enhanced suppression of proteins associated with pyroptosis. Meanwhile, ELISA analyses indicated that C-IVa-LPs-THRre substantially lowered IL-1β and IL-18 levels, thereby mitigating the inflammatory damage caused by pyroptosis.

### In vivo drug distribution studies

**Fig. 6:**
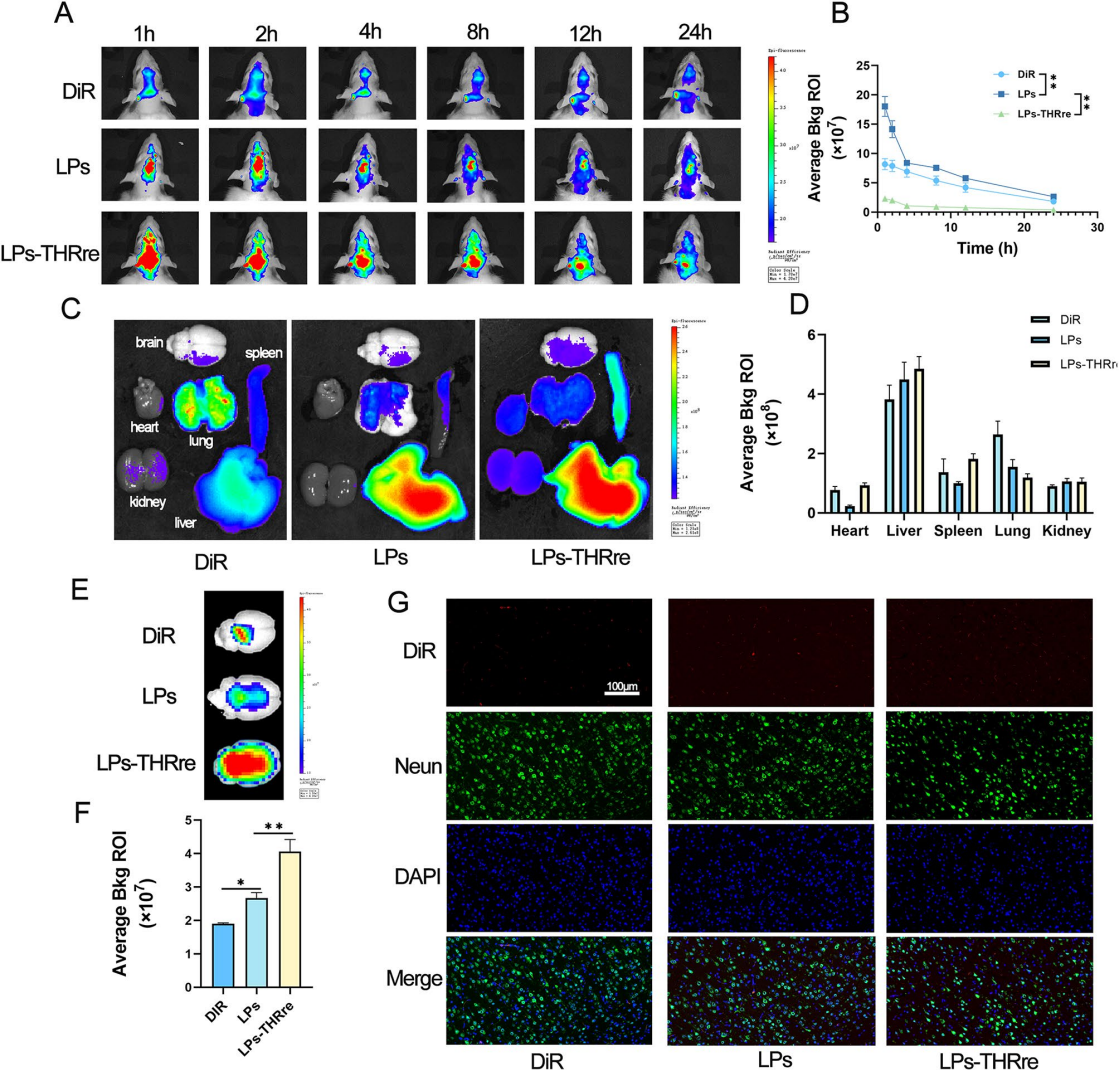
In vivo brain targeting of liposomes. (**A**) Fluorescence analysis of liposome distribution in brain tissue of the rats. (**B**) Quantitative fluorescence results in rat brain within 24 h (n = 3, ** *P*＜0.01, * *P*＜0.05). (**C**) Fluorescence analysis of liposome distribution in several main organs of rats, including the brain, heart, liver, spleen, lung, and kidney. (**D**) Quantitative fluorescence results of main organs (n = 3). (**E**) The mean fluorescence intensity in the brains of rats. (**F**) Quantitative results of fluorescence in isolated brain. (*n* = 3, ** *P* < 0.01; * *P* < 0.05) (**G**) Targeting of liposomes to neuron in the brains of rats (scale bar = 100 μm)

DiR-labeled liposomes were employed to monitor the in vivo drug distribution. As depicted in Fig. 6A&B, drug localization in rat brains was significantly greater in the two liposome groups compared to the monofluorescent group, with the DiR-LPs-THRre group showing superior accumulation. At 24 h post-administration, organs including the brain, heart, liver, spleen, lungs, and kidneys were excised to assess drug accumulation, as illustrated in Fig. 6C, and the fluorescence quantitative results are shown in Fig. 6D. Figure 6E&F presents isolated brain accumulation plots for each group, highlighting that notable drug residues remained in the brains of liposome-treated rats after 24 h, and modification with the THRre-peptide resulted in enhanced drug retention in the brain. To corroborate these findings, isolated brain tissues were extracted from rats 24 h following drug administration and subjected to cryosectioning to examine drug distribution within the brain. According to Fig. 6G, both liposome groups exhibited pronounced fluorescence signals, with the LPs-THRre group displaying markedly higher fluorescence intensity, suggesting that the THRre peptide significantly augmented drug accumulation in the rat brain.

### Evaluation of in vivo anti-AIS efficacy

**Fig. 7:**
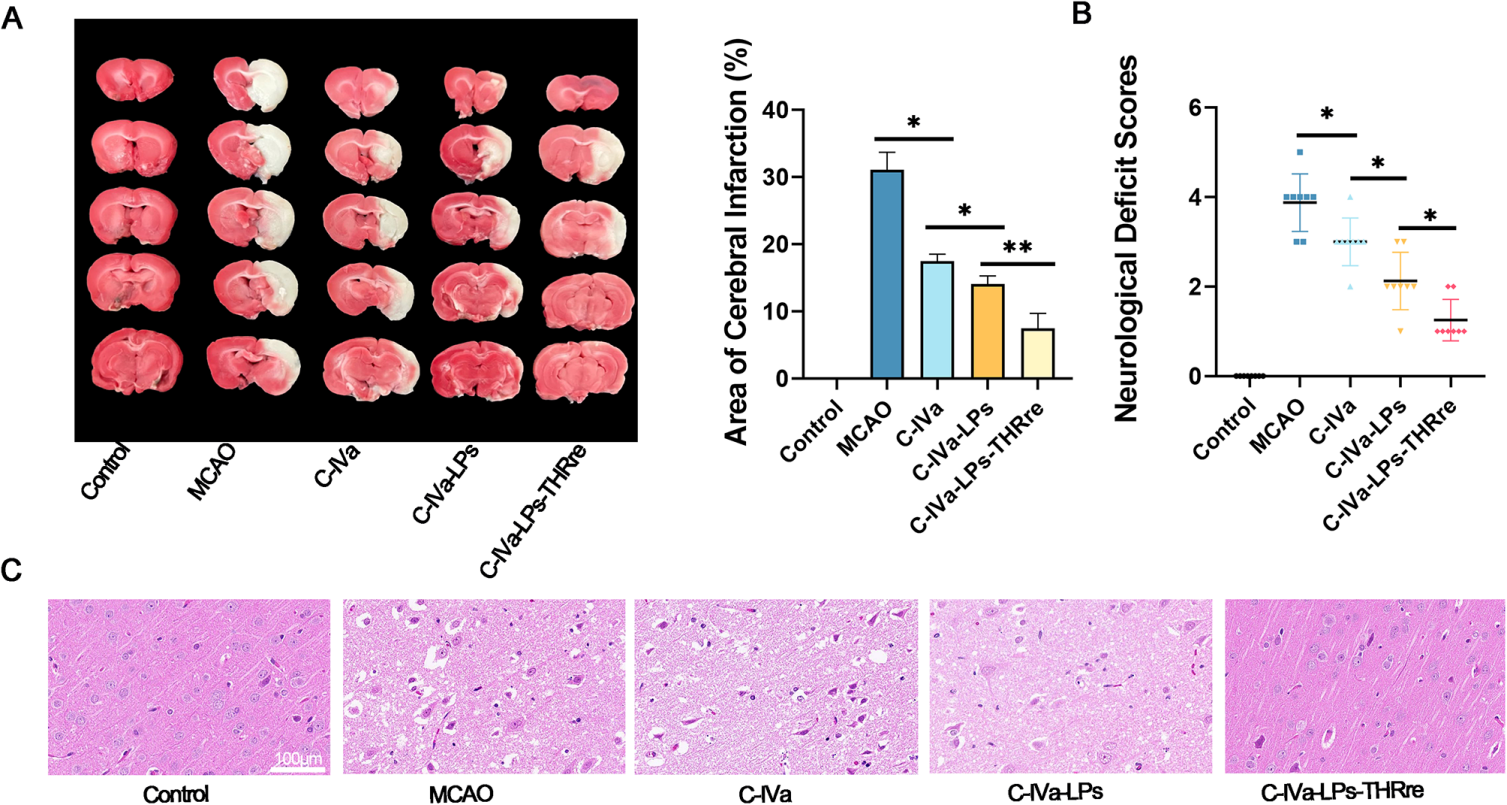
Neuroprotective effects of C-IVa-LPs-THRre on MCAO rats. (**A**) TTC staining and corresponding statistical analysis showed the effect of C-IVa-LPs-THRre on cerebral infarction volume. The red and white colors represent the normal and infarcted areas, respectively (*n* = 6). (**B**) Neurological Scores of MCAO rats (*n* = 8). (**C**) H&E staining was performed on the rat brains (scale bar = 100 μm) (** *P* < 0.01; * *P* < 0.05)

Neurofunctional scoring and assessment of cerebral infarction area were conducted 24 h post-drug administration in MCAO rats to preliminarily evaluate drug efficacy. As shown in Fig. 7A, the neurological deficits and cerebral infarct area were significantly increased in the MCAO group of rats. Compared to the group receiving free C-IVa, both liposome-treated groups exhibited substantial therapeutic effects. Specifically, the C-IVa-LPs-THRre group markedly improved neurological deficits and reduced the cerebral infarction area in rats.

The pathological alterations in rat brain tissue were examined using H&E staining. In the control group, brain tissue cells exhibited regular morphology, tight connections, and normal structural integrity. Conversely, cells in the MCAO group displayed disorganization and structural misalignment, characterized by enlarged intercellular spaces and edematous changes. Treatment with C-IVa-LPs-THRre significantly ameliorated these pathological features.

### Mechanism of C-IVa-LPs-THRre anti-pyroptosis in vivo

**Fig. 8:**
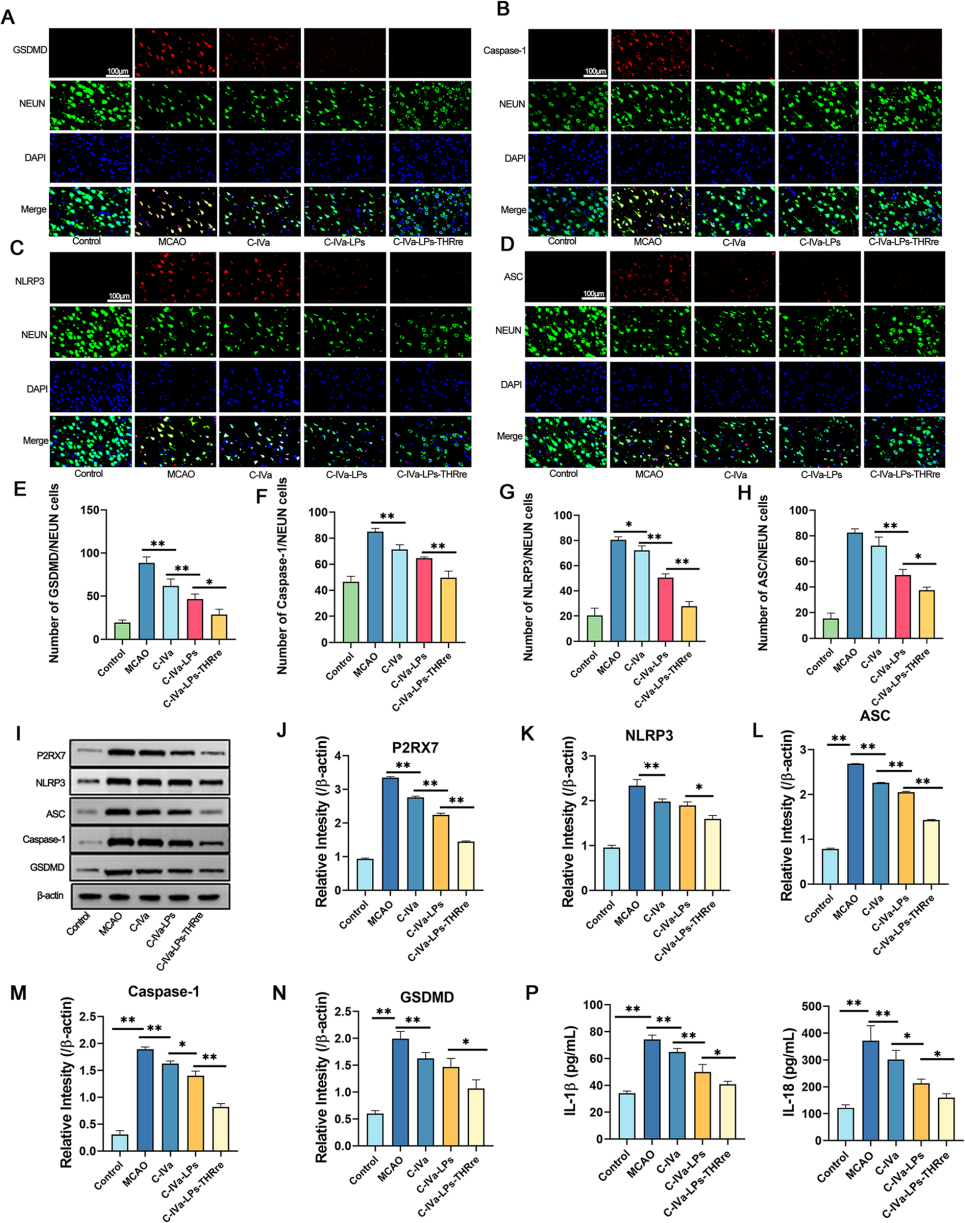
Effect of C-IVa-LPs-THRre on the expression of pyroptosis related proteins in brain tissue of MCAO rats. **A-D**. The expression levels of GSDMD, Caspase-1, NLRP3 and ASC based on immunofluorescent staining. **E-H**. Statistical analysis in the expression levels of GSDMD, Caspase-1, NLRP3 and ASC based on immunofluorescent staining. **I**. The expression levels of P2RX7, NLRP3, ASC, Caspase-1 and GSDMD were detected by Western blot. **J-N**. Statistical analysis in the expression levels of P2RX7, NLRP3, ASC, Caspase-1 and GSDMD. P. Effects of C-IVa-LPs-THRre on the expression levels of IL-1β and IL-18. (*n* = 3, ** *P* < 0.01; * *P* < 0.05)

Immunofluorescence was employed to assess the impact of C-IVa-LPs-THRre liposomes on the expression of pyroptosis-related proteins in the brain tissue of MCAO rats. Fig. 8A-H illustrate the expression levels of GSDMD, caspase-1, NLRP3, and ASC in localized neuronal cells. Both liposome groups exhibited a significant reduction in the expression of these proteins compared to the group treated with free C-IVa, with the C-IVa-LPs-THRre group showing a more pronounced effect than the C-IVa-LPs group. Western blot analysis (Fig. 8I-O) corroborated these findings, additionally revealing that C-IVa-LPs-THRre markedly decreased the expression of the P2RX7 receptor.

Subsequent to treatment, the expression levels of the inflammatory cytokines IL-1β and IL-18 were quantified in rat brain tissue using ELISA kits. As show in Fig. 8P, the findings indicated that administration of C-IVa-LPs-THRre to MCAO rats led to a significant reduction in IL-1β and IL-18 levels, thereby mitigating the inflammatory response.

## Discussion

The treatment of AIS continues to face numerous challenges. Current clinical strategies primarily focus on the removal of blood clots to restore cerebral circulation, however, this method presents a limited therapeutic window and carries a heightened risk of inducing cerebral hemorrhage [4]. Neuroprotective agents have become a hot spot in stroke research. A primary challenge for these agents, which are yet to be widely applied in stroke treatment, is the effective accumulation of drugs at ischemic sites and the optimal selection of the therapeutic time window [37]. The emergence of nanomedicines has begun to mitigate these issues. Liposomes, pivotal in the development of nanomedicine, offer distinct advantages and serve as versatile carriers for various targeted therapies currently in use.

Stroke disrupts the integrity of the BBB, with the release of large amounts of inflammatory mediators at the site of injury and the disruption of tight junctions between endothelial cells [38], which leads to high permeability of the BBB, a feature that can be leveraged to enhance drug accumulation in ischemic areas. Concurrently, liposomes have an enhanced permeability and retention effect that allows them to accumulate on the BBB, which is highly permeable to blood vessels [39]. Furthermore, it has been demonstrated that active targeting strategies, which utilize high-affinity ligands to target receptors highly expressed at the site, are effective regardless of changes in BBB permeability [40].

C-IVa has demonstrated potential as an anti-stroke neuroprotective agent in prior research; however, its mechanism against pyroptosis is not yet fully understood. In this study, we encapsulated C-IVa into liposomes for the first time, incorporating a retro-enantio isomer peptide, the THRre-peptide, to address the drug’s physicochemical limitations and enhance its delivery to the brain.

The particle size of our liposomes is less than 200 nm, aligning with the requirements for nanoformulations intended for brain delivery [41]. The targeting capability was enhanced by synthesizing DSPE-PEG-THRre to piggyback the THRre-peptide onto the liposomes. Subsequently, in vitro and in vivo toxicity evaluations demonstrated the safety and efficacy of liposomes. However, during the cytotoxicity evaluation, a marginally higher toxicity was observed in the THRre peptide-modified liposomes compared to the unmodified counterparts, which was speculated to be possibly due to incomplete purification during the synthesis of DSPE-PEG-THRre, coupled with enhanced cellular accumulation because of targeted delivery. On the other hand, no significant damage was observed in the H&E staining of organs in vivo, suggesting that animal metabolism may mitigate these effects.

The targeting properties of THRre-peptide were validated both in vivo and in vitro. A Transwell model was successfully constructed to simulate the BBB in vitro, assessing the BBB permeability facilitated by THRre-peptide. The results indicated that liposomes modified with THRre-peptide exhibited enhanced penetration through the BBB compared to unmodified liposomes. Cellular uptake experiments presented similar results and both reflected the time-dependence. These findings are consistent with the superior performance observed in vivo distribution experiments involving THRre-peptide-modified liposomes. However, in vivo assessments revealed that drug accumulation was not as time-dependent as in vitro cellular uptake, likely due to metabolic activities in vivo. Drug accumulation peaked at 1 h post-administration and subsequently declined gradually. Notably, the THRre-peptide group maintained a robust fluorescence signal at 24 h, potentially attributable to its resistance to protease hydrolysis.

We evaluated the pharmacodynamic performance of C-IVa-LPs-THRre both in vivo and in vitro in the context of stroke treatment. In vitro assessments revealed that although C-IVa-LPs-THRre exhibited increased toxicity in previously untreated cells, it effectively reversed the reduction in cellular activity in OGD/R-treated PC-12 cells. The MCAO model was established in SD rats, we chose to inject the drug within 0.5 h of reperfusion. Previous studies have shown that early (0.5 and 4 h) and delayed (24 and 48 h) after stroke were the optimal time points for intravenous delivery of liposomes to cover different stages of BBB injury. Liposome accumulation was significantly enhanced when injections were made at 0.5 and 48 h post-reperfusion [19]. THRre-peptide-modified liposomes significantly reduced cerebral infarct volume, and H&E staining confirmed their protective effects on cerebral cells.

C-IVa-LPs-THRre showed promising cerebroprotective effects both in vivo and in vitro, prompting further investigation into its impact on neuronal inflammation and pyroptosis. Some studies have reported that the P2RX7/NLRP3 pathway caused neuronal pyroptosis after ischemic stroke in mice [42]. Molecular docking studies involving C-IVa and proteins of the P2RX7/NLRP3/caspase-1 pathway revealed that C-IVa selectively binds to GSDMD and ASC (Supplementary Fig. 2), and the specificity of C-IVa’s interaction with other proteins remains unclear. It is hypothesized that C-IVa may affect the upstream and downstream molecules, potentially altering the expression of P2RX7, NLRP3, and Caspase-1 proteins. GSDMD is one of the hallmark proteins of pyroptosis [43] and ASC is a junction protein of inflammatory vesicles [44], the direct binding of C-IVa to these proteins substantiates its role in modulating cellular inflammation and the pyroptosis process. Experimental results from Western blot and ELISA analyses, as well as in vivo immunofluorescence staining, showed that C-IVa reduced the expression of pyroptosis proteins and downstream inflammatory factors, whereas C-IVa-LPs-THRre showed stronger activity, which fully demonstrated that THRre peptide-modified C-IVa brain-targeted liposomes effectively inhibit pyroptosis and mitigate neuroinflammatory responses induced by stroke.

This study has several limitations. Firstly, we did not investigate the impact of ligand concentration on drug accumulation, and there is currently no consensus on the peptide quantification necessary for effective BBB penetration [45]. However, previous research has demonstrated that small concentrations of ligands can exert significant effects [46]. *To reduce uncertainty and more accurately assess the contribution of the THRre peptide to drug delivery, we substituted DSPE-PEG with an equivalent mass. Our findings indicated that the incorporation of THRre peptide did not significantly influence particle size or encapsulation efficiency. Secondly, while PC-12 cells are a commonly utilized neuronal cell line, the complexity of neuronal cells in brain tissue suggests that primary neuronal cells may be more appropriate for such research. In future studies, we plan to investigate the impact of peptide concentration on brain-targeting efficiency and utilize primary neurons to further assess the neuroprotective effects of C-IVa liposomes modified with THRre-peptide on ischemic brain tissue. Finally, although our expertise in MCAO model preparation is advanced, in the preparation of our model, the introduction of the treated nylon thread from the CCA into the ICA during model preparation and the limited blood flow during reperfusion contributed to a relatively high mortality rate, which could introduce some bias. Despite these limitations, the validity of our conclusions has not be undermined.*

## Conclusion

In summary, we developed a liposomal drug delivery system encapsulating C-IVa and incorporates a retro-enantio peptide, THRre-peptide, specifically designed to target the brain and enhance anti-AIS efficacy. Our findings indicate that the inclusion of the THRre-peptide significantly increased cellular uptake in vitro and notably enhanced drug accumulation in the rat brain. The pharmacodynamic evaluation showed that C-IVa-LPs-THRre could significantly increase the survival rate of PC-12 cells, and in vivo, experiments indicated that it could significantly reduce the area of cerebral infarction and exert cerebroprotective effects. Mechanistic investigations suggested that C-IVa-LPs-THRre markedly reduced the expression of proteins within the P2RX7/NLRP3/Caspase-1 pathway, as well as associated inflammatory factors, thereby mitigating the inflammatory response and inhibiting pyroptosis induced by AIS.

## Electronic supplementary material

Below is the link to the electronic supplementary material.

Supplementary Material 1

## Data Availability

No datasets were generated or analysed during the current study.
