# Correction to: Chikusetsu saponin IVa liposomes modified with a retro-enantio peptide penetrating the blood-brain barrier to suppress pyroptosis in acute ischemic stroke rats

**DOI:** 10.1186/s12951-025-04017-2

**Published:** 2026-04-08

**Authors:** Yitong Liang, Tingting Fan, Min Bai, Na Cui, Wangting Li, Jingwen Wang, Yue Guan

**Affiliations:** 1 Department of Pharmacy, Xijing Hospital, Air Force Medical University, Changle West Road 127, Xi’an, Shaanxi, China; 2 Department of Geriatrics, Xijing Hospital, Air Force Medical University, Changle West Road 127, Xi’an, Shaanxi, China

In this article, during figure preparation, the author identified that in Fig. 6G, the cryosectioning image representing the LPs-THRre group was incorrectly displayed. The correct cryosectioning image for the LPs-THRre group in Fig. 6G has now been corrected in the original publication. For completeness and transparency, the correct and incorrect versions are displayed below.

Incorrect Fig. 6.

**Figure:**
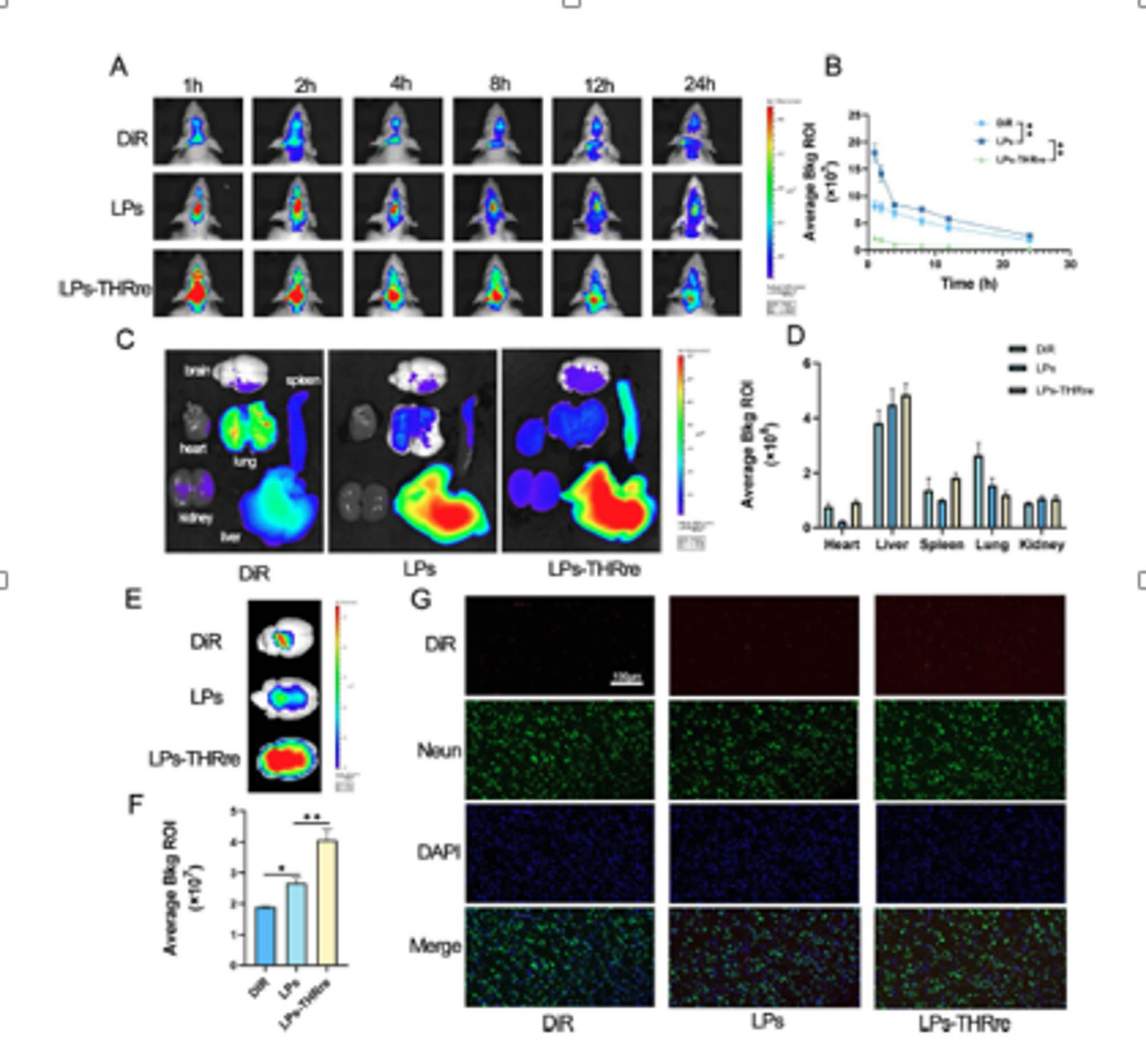
In vivo brain targeting of liposomes. (**A**) Fluorescence analysis of liposome distribution in brain tissue of the rats. (**B**) Quantitative fluorescence results in rat brain within 24 h (n = 3, ** P＜0.01, * P＜0.05). (**C**) Fluorescence analysis of liposome distribution in several main organs of rats, including the brain, heart, liver, spleen, lung, and kidney. (**D**) Quantitative fluorescence results of main organs (n = 3). (**E**) The mean fluorescence intensity in the brains of rats. (**F**) Quantitative results of fluorescence in isolated brain. (n = 3, ** P < 0.01; * P < 0.05) (**G**) Targeting of liposomes to neuron in the brains of rats (scale bar = 100 μm) Targeting of liposomes to neuron in the brains of rats (scale bar = 100 μm)

Correct Fig. 6.

**Figure:**
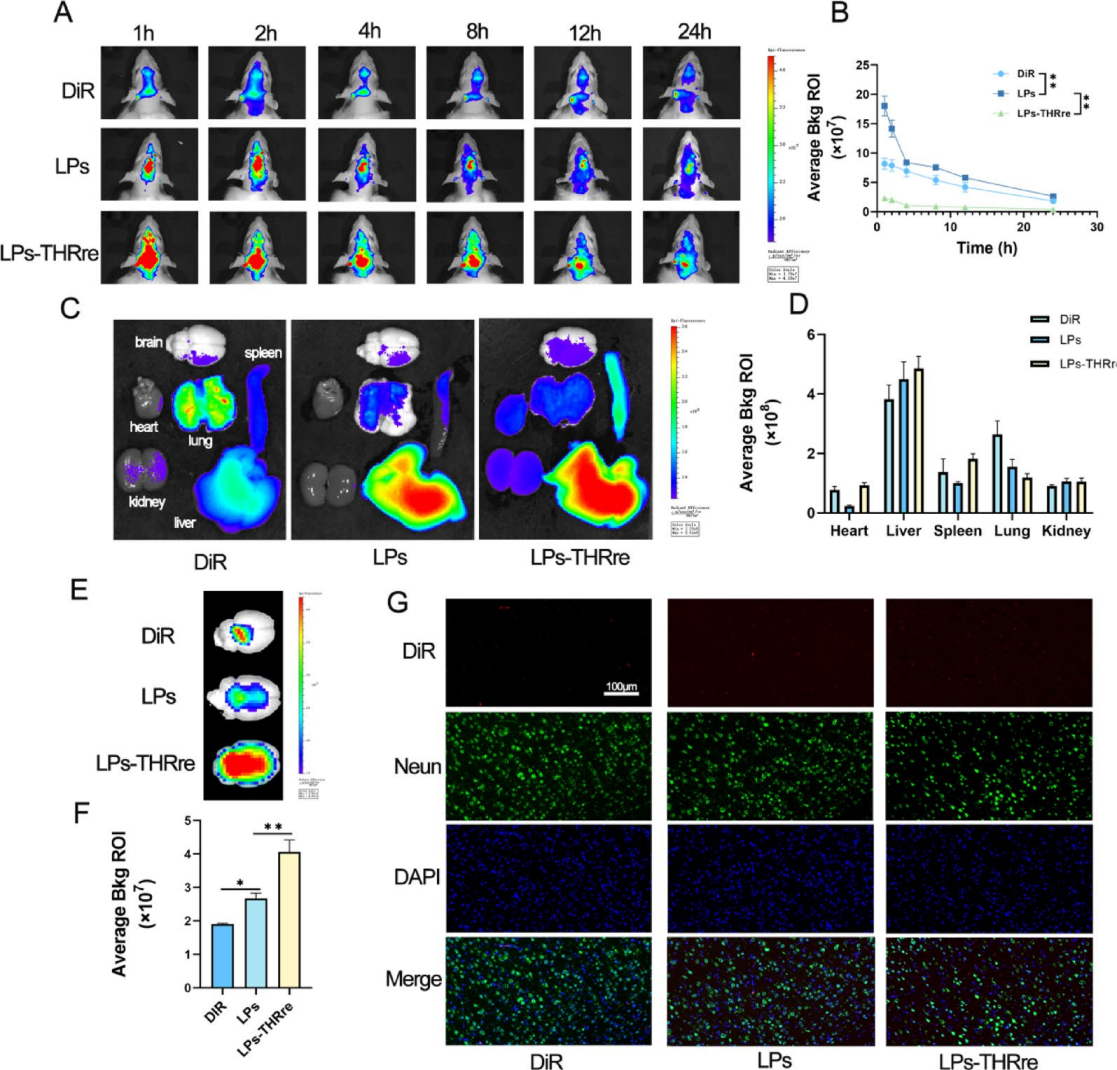
In vivo brain targeting of liposomes. (**A**) Fluorescence analysis of liposome distribution in brain tissue of the rats. (**B**) Quantitative fluorescence results in rat brain within 24 h (n = 3, ** P＜0.01, * P＜0.05). (**C**) Fluorescence analysis of liposome distribution in several main organs of rats, including the brain, heart, liver, spleen, lung, and kidney. (**D**) Quantitative fluorescence results of main organs (n = 3). (**E**) The mean fluorescence intensity in the brains of rats. (**F**) Quantitative results of fluorescence in isolated brain. (n = 3, ** P < 0.01; * P < 0.05) (**G**) Targeting of liposomes to neuron in the brains of rats (scale bar = 100 μm) Targeting of liposomes to neuron in the brains of rats (scale bar = 100 μm)

The original article has been corrected.

