# Supplementary material for: Chikusetsu Saponin IVa liposomes modified with a retro-enantio peptide penetrating the blood-brain barrier to suppress pyroptosis in acute ischemic stroke rats
Source: J Nanobiotechnology. 2024 Jul 4;22:393. doi: 10.1186/s12951-024-02641-y (PMC11223377; doi:10.1186/s12951-024-02641-y)
Supplement: Supplementary file 1 — Supplementary Material 1 [file 12951_2024_2641_MOESM1_ESM.docx]

**Supplementary material**

**Supplementary Figure**

**
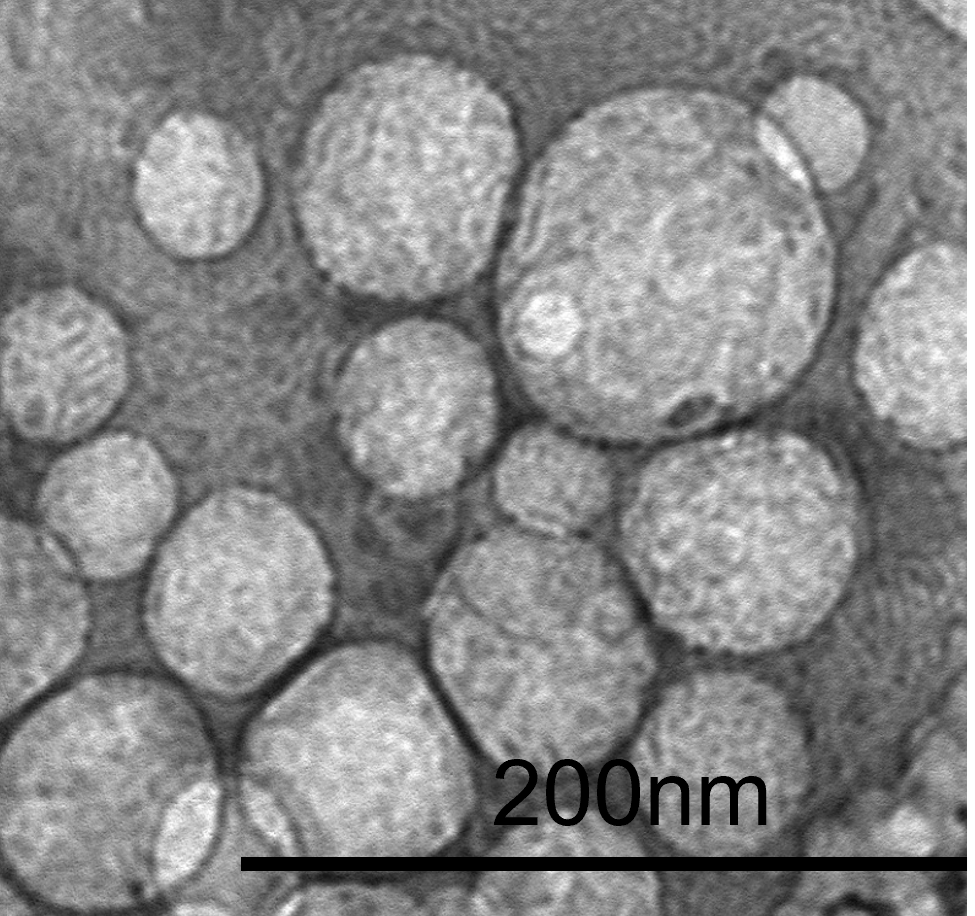
**

Supplementary Figure1 TEM images of LPs

A.
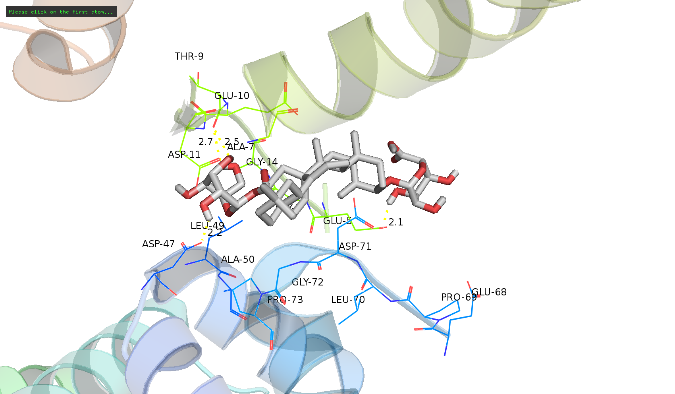
B.
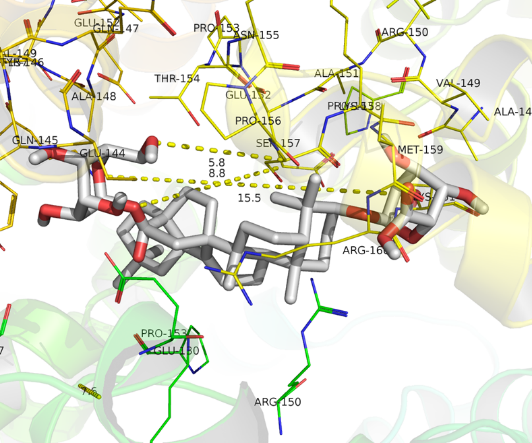


Supplementary Figure 2 Molecular docking diagram of C-IVa with GSDMD (A) and ASC (B).

**Supplementary Table**

Supplementary Table 1 Characterization of liposomes

|  | C-IVa-LP | C-IVa-LP-THRre |
| --- | --- | --- |
| EE% | 77.67±0.536 | 76.05±0.678 |
| DL% | 12.345±0.849 | 12.042±0.142 |
| size | 119.3±0.21 | 122.3±2.65 |
| PDI | 0.182±0.021 | 0.272±0.015 |
| ζ-potential | -25.4±0.95 | -20.5±3.66 |
